# Coarse needle surface potentiates analgesic effect elicited by acupuncture with twirling manipulation in rats with nociceptive pain

**DOI:** 10.1186/s12906-016-1505-2

**Published:** 2017-01-03

**Authors:** Sunoh Kwon, Yangseok Lee, Hi-Joon Park, Dae-Hyun Hahm

**Affiliations:** 1 Acupuncture and Meridian Science Research Center, College of Korean Medicine, Kyung Hee University, 26, Kyungheedae-ro, Dongdaemun-gu, Seoul, 02447 Republic of Korea; 2 Department of Basic Science of Korean Medicine, Graduate School, Kyung Hee University, 26, Kyungheedae-ro, Dongdaemun-gu, Seoul, 02447 Republic of Korea; 3 Department of Psychiatry and Behavioral Sciences, Northwestern University Feinberg School of Medicine, Chicago, 60611 USA; 4 KM Fundamental Research Division, Korea Institute of Oriental Medicine, Daejeon, 34054 Republic of Korea

**Keywords:** Acupuncture, Manipulation, Needle surface roughness, Connective tissue, Analgesia

## Abstract

**Background:**

Biomechanical phenomenon called “needle grasp” through the winding of connective tissue has been proposed as an action mechanism of acupuncture manipulation. The aim of the present study is to verify whether the needle grasp force affects the pain-relieving activity of acupuncture in the tail-flick latency (TFL) and the rat paw formalin tests.

**Methods:**

In order to make different roughness on the acupuncture needle surface, the needles with 0.2 mm-diameter were scratched using silicon carbide sandpapers with the grit numbers of 600 (mild coarse) and 200 (extra coarse). The surface roughness and rotation-induced torque of the scratched needles were then measured by atomic force microscope and Acusensor®, respectively. Rat abdominal wall tissues including insertion site of acupuncture needle were excised after 5 unidirectional rotations of the needles having various degrees of roughness, and the morphological changes of connective tissues were analyzed using hematoxylin and eosin (H-E) staining. Finally, the effects of coarse needle surface on anti-nociception induced by twirling manipulation were tested in rat TFL and formalin test.

**Results:**

It was observed that the rougher the needle surface, the stronger the needle grasp force and thickness of subcutaneous connective tissue while rotating. TFL increased in proportion to surface roughness of the ground needles 10 min after acupuncture into the Zusanli acupoint (ST36) on rat’s legs. In the rat formalin test, the rougher needle also significantly exerted the larger analgesic effect during both early and late phases compared to non-ground normal needle.

**Conclusion:**

Surface roughness of the acupuncture needle enhanced an anti-nociceptive activity of acupuncture therapy in rats, which partially supports the mechanical signaling theory through connective tissues in acupuncture manipulation.

## Background

Acupuncture has become increasingly popular in the Western world as a therapy for a wide range of pain difficult to manage with conventional treatments [1], however the mechanisms related to the therapeutic effect of acupuncture remains largely unknown [2].

To achieve therapeutic effect, acupuncture needles are manually manipulated after their insertion into the body. Acupuncture manipulation typically consists of rapid rotation (back-and-forth or uni-direction) and/or pistoning (up-and-down motion) of the needle [3]. A characteristic symptoms and reactive phenomenon linked to acupuncture manipulation is known as *de qi*, widely considered essential to acupuncture’s therapeutic effect [4–6]. *De qi* emphasizes a sensory component experienced by the patient as an aching sensation in the area of the inserted needle as well as a teasing sensation through the inserted needle that the acupuncturist feels as if the tissue is contracting around the needle, such that there is increased resistance to further motion of the needle [1, 4, 5, 7].

The theory quoted for needle manipulation is that it is due to a contraction of skeletal muscle [6, 8, 9], however this theory has not been supported by quantitative data regarding muscle contraction. As an alternative, Langevin et al. have previously hypothesized that a different and novel mechanism for needle grasp might involve the contraction of connective tissue, and proposed that the winding connective tissue during needle rotation creates a tight mechanical coupling between needle and tissue, which might allow needle manipulation to deliver a powerful mechanical signal into the tissue [10]. This hypothesis was supported by histological observations in rat tissue explants that showed marked thickening of subcutaneous tissue and a whorl of dense connective tissue around the rotated needle [10]. Whereas the importance of grasp force by winding connective tissues was elucidated by showing the morphological changes of tissues or neighboring cells around the pricking point of acupuncture manipulation in an ex vivo system, there have been no reports precisely explaining how the friction-induced grasp force between the needle surface and the contacting tissues correlates with alleviating the symptoms of diseases.

Manual manipulation of the needle (e.g., rotation, or pistoning) is used to clinically enhance needle grasp [4], and the needle grasp can be quantified by measuring the amount of force necessary to pull the acupuncture needle out of the skin (pullout force) [11]. We have previously reported that both twirling and lifting–thrusting manipulation potentiated acupuncture at acupoint ST36-induced analgesic effect in formalin-induced rats and that twirling had the more potentiation rather than lifting–thrusting [12]. Taken together, we hypothesized that twirling-induced frictional force between needle surface and adjacent tissues might be increased as the surface roughness of the acupuncture needle becomes coarser and that this frictional force affects acupuncture-elicited analgesic effect in rats with nociception. Thus, we used silicon carbide sandpapers with different grit numbers to manipulate the needle grasp force by changing surface roughness. We confirmed the different surface roughness and rotation-induced torques of the scratched acupuncture needles using atomic force microscope and Acusensor®, respectively. Under the various conditions of grasp force, it was also investigated whether the rotation manipulation of acupuncture with coarser surface at acupoint ST36 has more analgesic effects on tail-flick latency (TFL) test and formalin-induced pain behavior.

## Methods

### Animals

Male Sprague–Dawley rats, weighing 200–250 g, were purchased from Samtaco Animal Co. (Osan, Kyungki-do, Korea). All rats were housed in a limited access rodent facility with up to five rats per polycarbonate cage. The room controls were set to maintain the temperature at 22 ± 2 °C and the relative humidity at 55 ± 15%, the cages were lit by artificial light for 12 h each day, and sterilized drinking water and a standard chow diet were supplied ad libitum throughout the study. All animal experiments began a minimum of 7 days after the animals arrived, were conducted in accordance with the *Guide for the Care and Use of Laboratory Animals Eighth Edition* (by the National Research Council of the National Academies, revised in 2011), and were approved by the Kyung Hee University Institutional Animal Care and Use Committee. All efforts were made to minimize the number and suffering of animals.

### Generation of different surface roughness of acupuncture needle

A disposable stainless steel needle (silicone coated, ø0.20 × 60 mm) was purchased from Dongbang Acupuncture Ltd. (Kyunggi-do, Korea). The total length of the needle was 60 mm from which the handle part was 20 mm and the body part was 40 mm. In order to produce different surface roughness, premium silicon carbide discs having the grit numbers of 200 (80 μm) and 600 (15 μm) (3 M Korea Co., Seoul, Korea) was used to grind the surface of the body part. The scrapes on the needle surface were generated by pulling once as holding the handle part while the body part was strongly embedded by sandpaper, parallel to the needle axis. The scratch was confirmed by observing distal end of the needle tip using a microscope (BX51; Olympus Ltd., Tokyo, Japan), and subsequently photographed to compare their uniformity (200×).

### Atomic force microscope (AFM) observation of acupuncture needle surface

The AFM images were obtained using Nanostation II™ (Surface Imaging Systems, Herzogenrath, Germany) in non-contact mode. The Nanostation II™ was equipped with 92.5 μm XY/6 μm Z scanner and an optical microscope, Zeiss Epiplan 50× (Carl Zeiss, Oberkochen, Germany). The AFM was placed on top of the active vibration isolation table (TS-150, S.I.S., Herzogenrath, Germany), which was located inside of the passive vibration isolation table (Pucotech., Seoul, Korea) to eliminate external noise such as vibration. Data acquisition and processing were performed by the SPIP™ (Scanning Probe Image Processor, version 4.1, Image Metrology, Denmark). The reflex coated silicon cantilevers for non-contact mode (PR-NC, S.I.S., Germany) had the following characteristics: (manufacturer’s specifications: F = 146 ~ 236 kHz, C = 21-98 N/m, L = 225 μm and R = 0.01 ~ 0.02 Ohm · cm). Samples were scanned at the resolution of 256 × 256 pixel with scan speed of 1 line/s.

### Rotation-elicited torque measurement of acupuncture needle using Acusensor®

The measurement of acupuncture needle torque was performed using Acusensor® (Stromatec, Burlington, VT, USA) according to the protocol by RT Davis, DL Churchill, GJ Badger, J Dunn and HM Langevin [3]. Acusensor measurement system consists of two units: a needle motion sensor and a needle force (torque) sensor. Both sensors were cooperatively operated to quantitatively analyze rotational (back-and forth or one direction) and/or pistoning (up-and-down motion) manipulations. After the acupuncture needle was inserted at a depth of 5 mm perpendicularly to the skin using insertion tube of the needle motion sensor, the needle was manually rotated 3 times in one direction and the torque was measured in every 360° clockwise rotation.

### Experimental groups and acupuncture treatment

Animals were randomly divided into four treatment groups: the normal group without any treatments (NOR, *n* = 7), the plain acupuncture group without scraping of needle surface (ACU, *n* = 7), the acupuncture group with needle surface of low roughness (ACU600, *n* = 7), the acupuncture group with needle surface of high roughness (ACU200, *n* = 7), and morphine (10 mg/kg, *i.p*.)-treated group (MOR10, *n* = 7).

TFL test and formalin test were performed immediately after the acupuncture treatment or morphine administration. Morphine was used as the positive control and its dose was adopted according to the previous study [13]. The needle was inserted into the acupoint ST36 on the dextral side at a depth of 5 mm and then manually twirled 360° clockwise and counterclockwise as one cycle, three cycles per second for the total duration of three seconds. The acupoint ST36 is located at the proximal one fifth point on the line from the depression lateral to the patella ligament to the anterior side of ankle [14].

### Histochemical staining of abdominal skin tissue

**Fig. 1:**
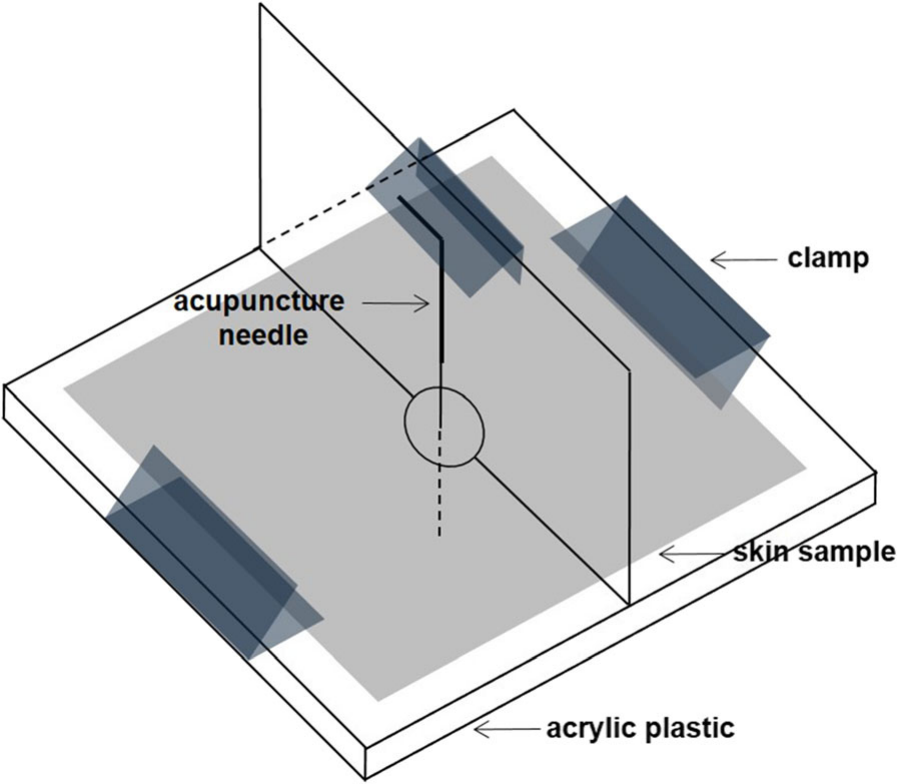
Schematic diagram of the equipment to tightly hold and spread the skin tissue and to fix an acupuncture needle inserted into the skin perpendicularly and rotated uni-directionally

In order to verify the histological changes of the abdominal skin layers including epidermis, dermis, subcutaneous tissue and abdominal wall muscle, caused by acupuncture needle twisting, an acrylic equipment was designed tightly to hold the skin explant. While anesthetizing with a sodium pentobarbital (80 mg/kg, i.p.), rat abdominal skin including the skin abdominal wall, with a liberal margin of surrounding skin, was excised to a depth to include the underlying connective tissue above the external fascia of the dorsal muscles wall with an appropriate size (30 mm × 30 mm) using a surgical knife, washed in phosphate buffered saline (PBS) for 5 min, and tied up to the acrylic plastic frame using clamps. Subsequently the needles with various surface roughness were inserted at a depth of 5 mm into the ex vivo abdominal skin and uni-directionally rotated 5 consecutive cycles. The schematic of the needle rotation for histology with the acupuncture needle inserted to the abdominal skin is depicted in Fig. 1. We picked up the abdominal skin tissues instead of the anterior tibialis skin tissues (ST36 region) to observe the morphological intorsion of connective tissue induced by twirling manipulation. The abdominal tissues are wider and softer, which helps to execute tissue biopsy easily and to maximize the visualization, whereas the anterior tibialis tissue is too narrow to cut off so it was impossible to show the intorsion due to technical limitation. Nevertheless, we can suppose the morphological change by twirling manipulation might be occurred in the anterior tibialis tissue as the same aspect in the abdominal tissue, although we cannot observe visually.

These biopsy specimens were fixed in 4% paraformaldehyde overnight, dehydrated through a graded ethanol series, embedded in paraffin, sectioned parallel to the needle axis at 10 μm thickness using a rotatory microtome, Shandon Finesse 325 (Thermo Fisher Scientific Inc., MA USA), and mounted on slides. Before staining, slides were deparaffinized. For demonstrating morphologic changes and eosinophil infiltration, the slides were stained with hematoxylin (Merck Co., Darmstadt, Germany) and 1% eosin (Sigma-Aldrich Co., St. Louise, MO, USA). The slides were bathed in hematioxylin for 7 min, distilled water for 5 min, 1% HCL tapping for 3 times, 80% ethanol (EtOH) for 3 min, 100% EtOH for 3 min, 1% eosin for 1 min, 80% EtOH for 1 min, 90% EtOH for 5 min and 100% EtOH for 5 min, continuingly. The slides were finally put into 100% xylene for 3 min and this procedure was repeated three times. Then, 2–3 drops of permount were dropped directly to the tissues on the slide, and a cover slip was gently placed over the tissues after pressing out the bubbles with tweezers. These slides were air-dried and cover-slipped for microscopic observation. All slides (40× magnification) were observed, photographed using a microscope (BX51; Olympus Ltd., Tokyo, Japan).

### Tail flick latency and formalin tests

Anti-nociception was assessed using TFL and the rat formalin tests. TFL test was conducted using a model 33 tail flick analgesia meter (IITC Life Science Inc., Woodland Hills, CA) with the beam intensity set at 4.0 (Fig. 5.). All rats were habituated for 30 minutes in the procedure room prior to testing. During the TFL, rats were wrapped with a soft paper towel with the whole tail length exposed, and handheld with appropriate strength.

To perform the formalin test, 50 μL of 5% formalin was injected subcutaneously into the plantar surface of the right hind paw with a 30-gauge needle, then pain behaviors of the rats were examined for 60 min after formalin injection. Nociceptive behaviors were quantified by counting the number of times the animal licked, bit, or shaked the formalin-injected paw at 5-min intervals. Two phases of spontaneous nociceptive behavior were observed: an initial acute phase (early phase, duration of the first 10 min after formalin injection) was followed by a relative short quiescent period and then by a prolonged tonic response (late phase, duration of 50 min after the early phase). The analgesic effect of acupuncture with rough needle surface was compared with that of morphine (10 mg/kg, *i.p*.), an opioid analgesic drug, in both tests.

### Statistical analysis

The experimental results were expressed as the mean ± standard error (SEM). The behavioral data were calculated and analyzed by repeated measures analysis of variance (ANOVA) and one-way ANOVA followed by Tukey’s *post hoc* test using SPSS (Version 13.0; SPSS Inc., Chicago, USA). In all analyses, *p* < 0.05 was considered significant.

## Results

### Generation of different surface roughness of acupuncture needle surface

**Fig. 2:**
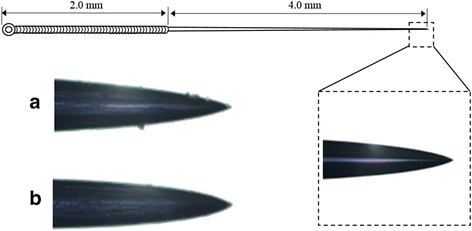
Roughness of acupuncture needles. The surface roughness was observed using a microscope (SZ61, Olympus Co., Japan). The tips of the needles were presented in the photos. **a** and **b** indicate acupuncture needles with high (200 grit number) and low (600 grit number) coarseness, respectively. The lengths of the needle handle and body were 20 mm and 40 mm, respectively

In order to increase the grasp force of needle differently, silicon carbide sandpapers with different grit numbers were used to make different longitudinal scratches on the needle surfaces. As shown in Fig. 2, long scratches were evidently observed on the ground needle surface along the needle axis and even some splinters in case of extra coarse sandpaper (grit number 200 in ACU200) while the surface of normal needle tip was observed slick and smooth.

### Atomic force microscope (AFM) observation of scratched acupuncture needle surface

**Fig. 3:**
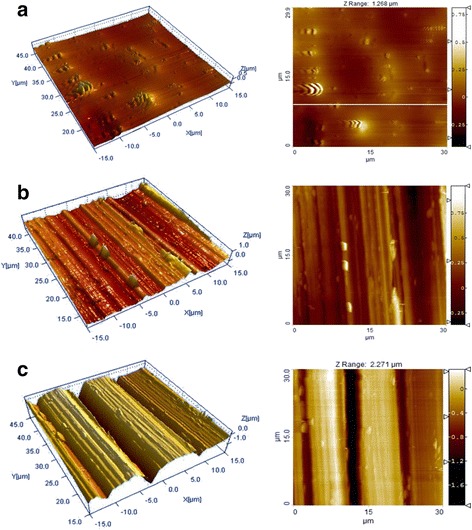
The different shapes of the scratches on the differently scraped needle surfaces in the stereomicroscope images were obtained by using atomic force microscope (AFM). The normal (ACU, **a**), lightly scraped (ACU600, **b**), and deeply scraped (ACU200, **c**) acupuncture needles

**Fig. 4:**
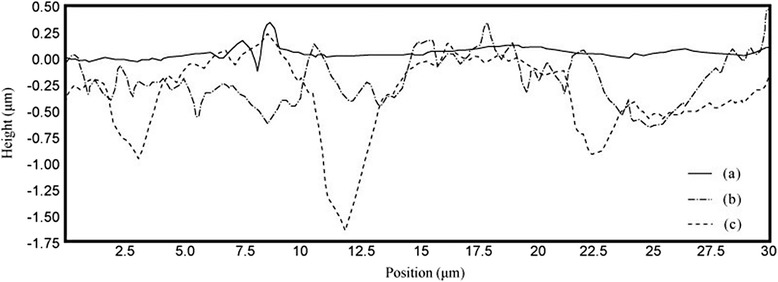
The different depths of abrasions on the differently scraped needle surfaces were observed. The maximum depth were about 0.625 μm in lightly scraped and 1.625 μm in deeply scraped, respectively. The normal (solid line, a), lightly scraped (dash-dotted line, b) and deeply scraped (dashed line, c) needle surfaces

AFM is one of the foremost imaging tools by which we can measure and manipulate matters at the nanoscale by using a high-resolution scanning probe (tip), and it is generally used to scan the specimen surfaces. In order to verify the scratches on the needle surface analytically, the shape and depth of the scratch were analyzed using an AFM. Stereomicroscopic images of normal, lightly scraped and deeply scraped needle surfaces were shown in Fig. 3. The surface of normal needle was flat and smooth even though there were several small bumps with various sizes but less than 0.5 μm height. Lightly scraped needle obviously showed the stripe-shaped artificial scratches with about 2 μm interval whereas deeply scraped needle had the deeper scratches with about 10 μm interval. Most of surfaces of scraped needles had significant abrasion compared with the smooth surface of normal needle as shown in Fig. 4. The maximum depth of the scratches on the surfaces of lightly scraped and deeply scraped needles were about 0.625 and 1.625 μm, respectively even though the patterns of scratches of both needles were irregular.

### Measurement of twirling-elicited torque in scratched acupuncture needle using Acusensor®

**Fig. 5:**
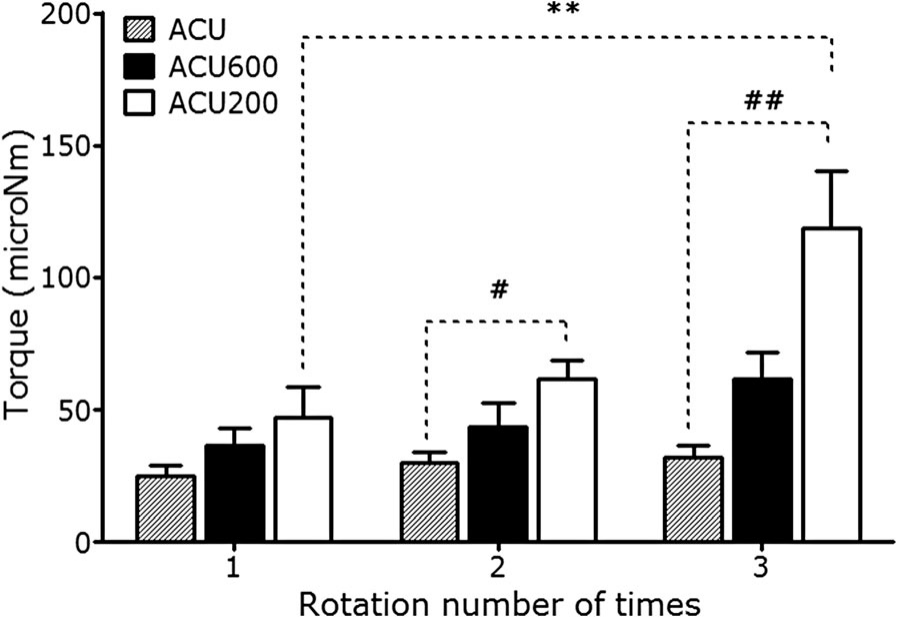
Acupuncture needle torque developed by skin tissue resistance against uni-directional rotation of the needle (total three times of rotation) were measured by using Acusensor®. Manipulation of deeply scraped needle (ACU200) significantly increased the torque as the rotation number increased. ^**^ *p* < 0.01 versus one time of rotation; ^#^ *p* < 0.05, ^##^ *p* < 0.01 versus ACU group

In order to quantify acupuncture needle manipulation according to the rotation number in uni-direction, Acusensor*®* was used for measuring the torque, a mechanical load developed by skin tissue that has tendency of resisting against the twirling needle. Uni-directional rotation of deeply scraped needles significantly increased torque as the rotation number was increased from 1 to 3. In the second and third rotation, the torque developed by rotating scraped needles was consistently greater than that by normal one, and the torque by deeply scraped needle was also greater that that by lightly scraped one (Fig. 5).

### Histochemical changes of abdominal skin tissues after twirling manipulation of scratched acupuncture needle

**Fig. 6:**
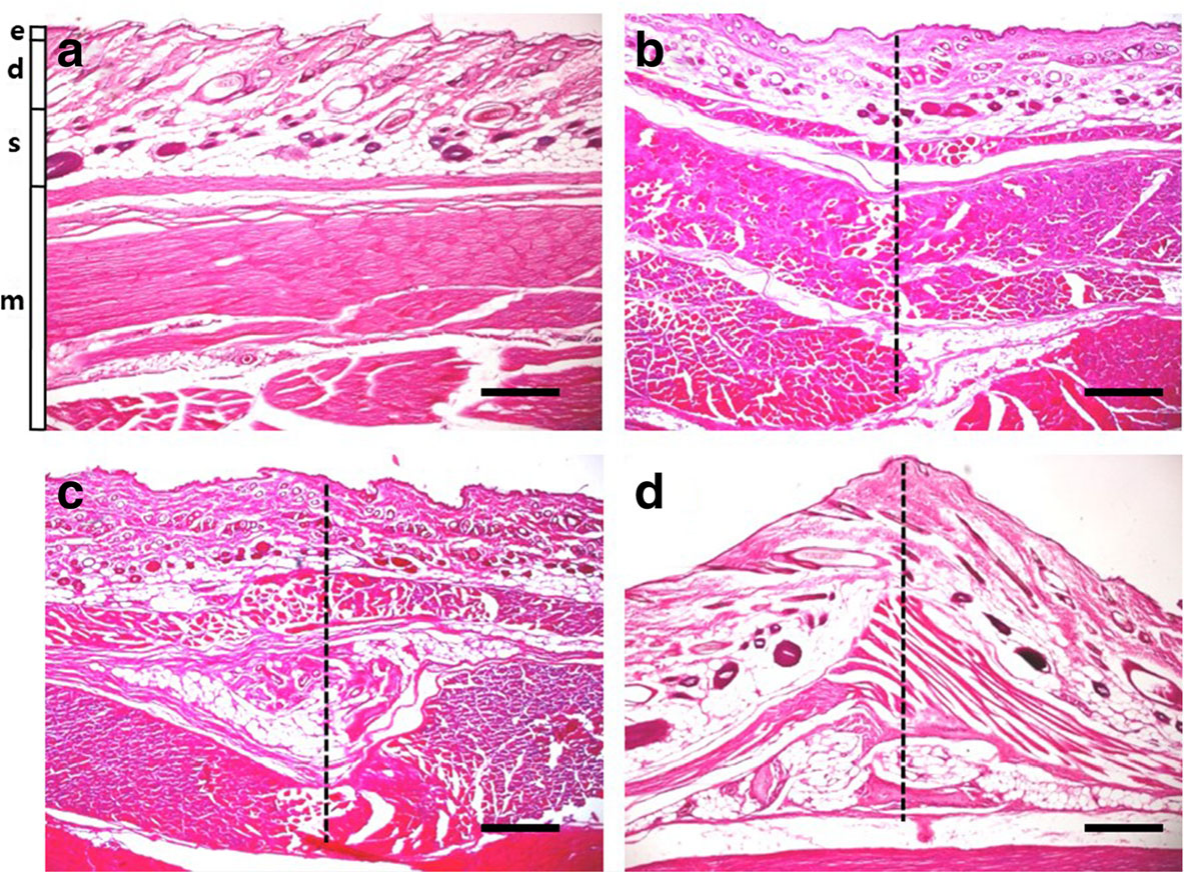
The different intorsion shapes of dermal tissues induced by twirling manipulation with the differently scraped needles were observed by using hematoxylin-eosin (HE) staining of histological sections (40×) of abdominal skin tissues. Without manipulation-NOR (**a**) and after rotation manipulation of the needle with smooth surface-ACU (**b**), lightly scraped surface-ACU600 (**c**) and deeply scraped surface-ACU200 (**d**). Abdominal skin layers include epidermis (e), dermis (d), subcutaneous tissue (s) and abdominal muscle (m). The dotted line indicates the inserted trace of acupuncture needle. Scale bars, 100 μm

Histological examination of tissue sections revealed that acupuncture needle penetrated epidermis, dermis, subcutaneous and muscle layers of skin, and that marked rise and thickening of skin tissue layers were observed along the axis of the deeply scraped needle (Fig. 6d) whereas slight thickening and deformity of subcutaneous and muscle layers were observed in the vicinity of the lightly scraped needle (Fig. 6c). Deformity of dermal and subcutaneous layers harboring skin fibroblast cells was remarkable among the skin layers observed. In case of deeply scraped needle, arrangements of various cells and extracellular structures such as adipose tissue, connective tissue, hair follicle vein and artery were highly twisted and stretched (Fig. 6d).

### Analgesic effect of twirling manipulation of scratched acupuncture needle in tail flick latency and formalin test

**Fig. 7:**
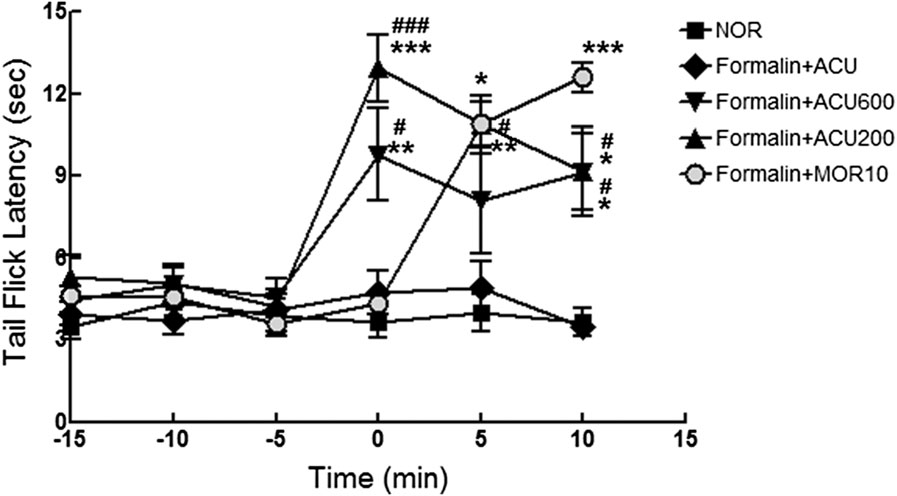
Tail flick latency (TFL) in rats treated with acupuncture needle with different roughness. Time zero denotes the onset of acupuncture treatment with rotation manipulation. Morphine was used as a positive control. NOR: non-treated normal group, Formalin + ACU: plain needle group, Formalin + ACU600: lightly scraped needle group, Formalin + ACU200: deeply scraped needle group, Formalin + MOR10: morphine group. ^*^ *p* < 0.05, ^**^ *p* < 0.01, ^***^ *p* < 0.001 versus NOR group; ^#^ *p* < 0.05, ^###^ *p* < 0.001 versus ACU group

After confirming the proportional increase of grasp force depending on surface roughness of the acupuncture needle, we subsequently investigated whether the strength of needle grasp force can affect the analgesic effect of acupuncture manipulation using TFL and the rat paw formalin tests in the rats. We observed that the rougher the needle surface, the stronger the pain relieving effect although the difference of analgesic activity between deeply scraped and lightly scraped needles was not statistically significant 10 min after acupuncture treatment (Fig. 7). These results may indicate that the pain-relieving efficacy induced by twirling manipulation was gradually decreased after reaching its peak at 10 min whereas intraperitoneal administration of morphine as a positive control was gradually increase analgesic effect. Twirling plain needle did not increase TFL, as compared with non-treated normal group.

**Fig. 8:**
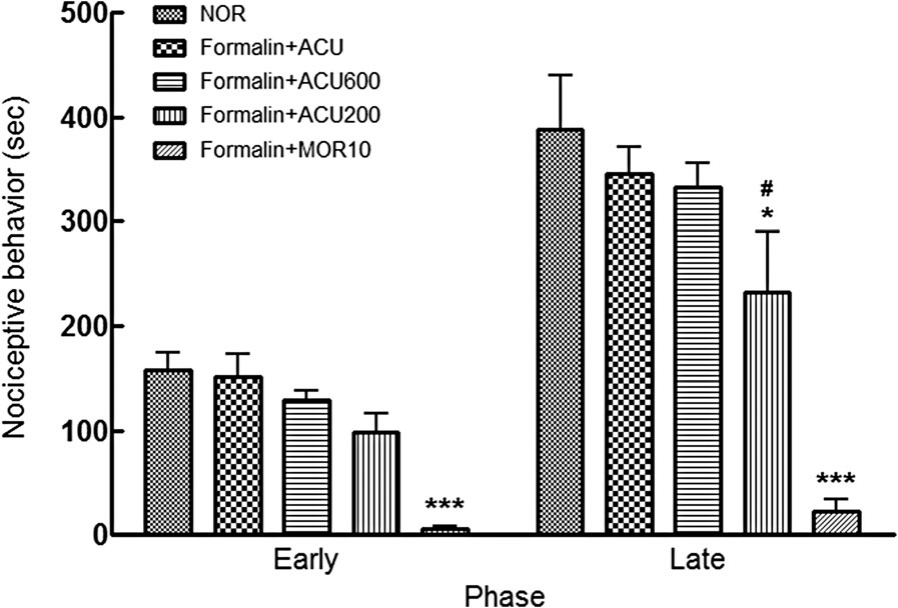
Pain behavior after formalin injection roughness-dependently in early and late phase. NOR: non-treated normal group, ACU: plain needle group, Formalin + ACU600: lightly scraped needle group, Formalin + ACU200: deeply scraped needle group, Formalin + MOR10: morphine group. ^*^ *p* < 0.05, ^***^ *p* < 0.001 versus NOR group; ^#^ *p* < 0.05 versus ACU group

The analgesic effect of twirling manipulation with coarse surface needle was also verified in the rat formalin test. In the early phase, there was a trend of decrease in nociceptive behaviors in coarse surface acupuncture-treated groups although it was not statistically significant (Fig. 8). In the late phase, nociceptive behaviors were significantly decreased in deeply scraped acupuncture-treated group (ACU200 group) as compared with those in non-treated normal group (NOR group). However, there were little differences in nociceptive behaviors between plain (ACU group) and lightly scraped (ACU600 group) acupuncture-treated groups. Formalin-induced nociceptive behaviors were almost removed by morphine injection both in the early and late phases.

## Discussion

In the present study, we investigated if the coarser needle surface of acupuncture induces the stronger needle grasp force by twirling manipulation and if anti-nociceptic effect of acupuncture becomes the more effective as the grater needle grasp force is occurred in distorted connective tissues. The main findings were that 1) acupuncture which has the deeper longitudinal scratches on the needle surface induced the grater needle grasp force in the skin tissue and that 2) the grater needle grasp force at acupoint ST36 showed the more effective anti-nociception.

An important aspect of acupuncture treatment is that acupuncture needle must be manually manipulated to achieve the best outcome after inserted into the body [1, 5]. Therefore, most of physicians have always used various manipulation techniques during acupuncture treatment, such as twirling and rotating, lifting and thrusting, flicking and scraping, and shaking and vibrating to boost up stimulating effect of acupuncture on the acupoint in addition to pricking effect of acupuncture needle [5, 15]. Among them, twirling manipulation, a finger skill of sequential order in a clockwise-counterclockwise manner after insertion of needle into the acupoint, has been the most popular because it is optimal way to adjust the acupuncture stimulation to get the *de qi* response. However, the underlying mechanisms of acupuncture manipulation and the signaling initiators or mediators generated by needle pricking and manipulation at the acupuncture point have remained unresolved. To understand its mechanism, the initiation of signaling on the peripheral acupuncture point on the skin due to acupuncture pricking and manipulation, and its long distance conveyance to the cognate internal organs should be necessarily investigated.

Traditionally or even in the present, the *de qi* sensation between acupuncturist and patient is the essential to clinically succeed acupuncture therapy. However this sensation is necessarily dependent on the emotional states of individual patients and their environmental atmosphere, and therefore great efforts have been made to scientifically understand and establish the relationship between the degree of *de qi* and its clinical efficacy [16]. Among them, Langevin’s group had assumed that the manipulation-associated acupuncturist’s *de qi* was due to the needle grasp force attributed to the increased resistance between the twirled needle and the distorted skin tissue as the number of times being uni-directionally rotated was increased. They addressed this biomechanical component of *de qi* experienced by acupuncturist as “needle grasp force” and suggested as a plausible mechanism of acupuncture manipulation [17].

If the direction and number of twirling are fixed, the needle grasp force will definitely depend on friction between needle surface and adjacent tissues, cells or extracellular structures surrounding the needle. We therefore proposed a hypothesis that scratching the needle surface may increase the resistance in the twirling manipulation. If the twirling manipulation can reinforce acupuncture therapy, surface roughness of needle will be a crucial parameter influencing therapeutic efficacy of acupuncture. Taken together, we can readily assume that the coarser the acupuncture needle surface is, the more effective therapeutic efficacy of acupuncture manipulation is.

In the present study, we used two different silicon carbide sandpapers to make different levels of surface roughness on the acupuncture needles: sandpapers having the grit numbers of 200 (extra coarse, ACU200) and 600 (mild coarse, ACU600), and confirmed the longitudinal deeper scratches along the needle axis and even some splinters in the needle ground with extra coarse sandpaper (grit No. 200). We also confirmed the degree of needle surface roughness is closely associated with the twirling-induced torque and distortion in skin tissue, which indicates the frictional force between needle surface and adjacent tissues may be increased as the surface roughness becomes coarser. Although Langevin’s group strongly formulated a grasp force hypothesis closely associated with winding of connective tissues as a biomechanical mechanism of twisting acupuncture manipulation, they have not suggested an experimental evidence proving that therapeutic effect of acupuncture manipulation is in accordance with the needle grasp force of skin tissues surrounding the inserted needle as of yet.

Thus, we investigated needle grasp force theory in nociceptive pain animal models, such as tail flick latency (TFL) test and the rat formalin test, to verify the analgesic activities of twirling manipulation using acupuncture needles with different surface roughness. TFL is a reflexive pain test designed to verify pain threshold against heat stimulus whereas the formalin test is a non-reflexive pain test, well-characterized tonic chemogenic pain model [18]. TFL was increased in proportion to the surface roughness of the acupuncture needles in the twirling of acupuncture. Acupuncture needle with rougher surface significantly exhibited larger analgesic effect during the late phases of the rat formalin test, as compared to that with smooth surface. These findings indicate the needle grasp force may be strongly associated with analgesic effect of twirling acupuncture manipulation against nociceptive pain.

We might be able to speculate the relationship between the needle grasp force and the analgesic effect from the review explaining the mechanistic and biological evidences of acupuncture manipulation. Acupuncture manipulation can induce the propagation of the acoustic wave and the response of calcium ion channel signaling, then the calcium ion channel-dependent peripheral secretion of endogenous opioids might subsequently follow, which might not show the addictive side effects [19]. Thus, it is possible that the increase in the dose of manipulation could get better analgesic effect because the stronger needle grasp force can induce the wider propagation of wave.

We could observe ACU200 showed anti-nociceptive effect in formalin test, nevertheless we did not figure out the reason that the ACU200 showed the effectiveness during the late phase only. The previous study showed manual acupuncture stimulation reversed nociceptive behavior during the late phase in the formalin test, which indicates acupuncture may be effective in relieving inflammatory pain rather than activation of peripheral nociceptors [20]. Moreover, the formalin response during the late phase is related to the CNS sensitization facilitated by the formalin-induced TRPA1 (Transient receptor potential cation channel, subfamily A, member 1) activation [21]. Taken together, we can guess ACU200 might be relieving inflammatory pain and modulating a part of TRPA1 signaling, which remains to be elucidated.

However, plain needle with twirling manipulation did now show significant analgesic effect in the present study. We previously reported formalin-induced pain was significantly alleviated by twirling acupuncture manipulation at acupoint ST36, but the number and total duration of manipulation cycle were ten times more than that in the present study [12]. This contradiction might be attributable to the reduced dose of manipulation. Because the discrepancy as four to five times in the torque induced by 3-cycle rotations between plain and extra coarse needle was found, we expected to get the grater stimulation from the coarser needle, which is the reason why we reduced the dose. In other words, we possibly get the *de qi* response with the much less quantity of manipulating coarser needle rather than plain needle.

The present study was designed to develop the optimal acupuncture device effective under the only manual stimulation for the shorter duration of manipulation. A lot of previous studies have shown the strong analgesic effect of electroacupuncture in the animal models of nociceptive pain, however electroacupuncture should be carefully considered by practitioners who take care of some patients (eg. unpleasant to electric stimuli, pacemaker user, etc.). Moreover, the best retaining-needle duration of manual acupuncture to get analgesic effect is suggested as 20 min [22], however the practitioners can experience the clinical case that they are not able to retain the acupuncture needle for such a long time. Therefore, we suggest the coarse needle surface acupuncture might be used to overcome those clinical difficulties. However, we recognize the limitations of the present study as the follows; 1) The scratches on the coarse needle surfaces of ACU200 and ACU600 were not even because we manually manufactured these needles as prototypes. This unevenness might occur the individual difference in the quantity of stimulation, so we need to find the way to improve quality control of the acupuncture needles. 2) Although we found the analgesic effect of the coarse acupuncture is comparable to the morphine, we did not unearth the specified mechanism which remains to be fully elucidated in the further study.

## Conclusions

Taken together, the twirling manipulation using coarser surface acupuncture needle significantly improved tonic and phasic pain better than plain surface needle, and eventually exhibited the more effective anti-nociceptive activity in rats. These results strongly support Langevin group’s mechanical signaling hypothesis of needle grasp force to elucidate the twirling acupuncture manipulation. Although we propose here a strategy for developing a novel acupuncture needle with coarser surface which probably produces stronger pain-relieving effect, a marked progress has not been achieved of yet and more investigations are required to be made in the future to elucidate molecular mechanism and all aspects of scientific mechanism of acupuncture therapy including manipulation.

## Abbreviations

AFM: Atomic force microscope
ANOVA: Analysis of variance
EtOH: Ethanol
H-E staining: Hematoxylin and eosin staining
TFL: Tail-flick latency
TRPA1: Transient receptor potential cation channel, subfamily A, member 1
